# DCE-MRI assessment of the effect of vandetanib on tumor vasculature in patients with advanced colorectal cancer and liver metastases: a randomized phase I study

**DOI:** 10.1186/2040-2384-1-5

**Published:** 2009-09-21

**Authors:** Klaus Mross, Ulrike Fasol, Annette Frost, Robin Benkelmann, Jan Kuhlmann, Martin Büchert, Clemens Unger, Hubert Blum, Jürgen Hennig, Tsveta P Milenkova, Jean Tessier, Annetta D Krebs, Anderson J Ryan, Richard Fischer

**Affiliations:** 1 Tumor Biology Centre at the Albert-Ludwigs-University, Freiburg, Germany; 2 Magnetic Resonance Development and Application Center, Universitätsklinikum, Freiburg, Germany; 3 Tumor Centre Ludwig-Heilmeyer, Comprehensive Cancer Center Freiburg, University Hospital, Freiburg, Germany; 4 AstraZeneca, Macclesfield, UK; 5 AstraZeneca, Wilmington, DE, USA

## Abstract

**Background:**

Vandetanib is a once-daily oral inhibitor of VEGFR, EGFR and RET signaling pathways. In patients with advanced colorectal cancer and liver metastases, the effect of vandetanib on tumor vasculature was assessed using dynamic contrast-enhanced magnetic resonance imaging (DCE-MRI).

**Methods:**

Eligible patients received vandetanib 100 or 300 mg/day. DCE-MRI (iAUC_60 _and K^trans^) was used to quantify the primary endpoints of tumor perfusion and vascular permeability. An exploratory assessment of tumor oxygenation was performed using MRI/T2*. All MRI parameters were measured at baseline (twice) and on days 2, 8, 29 and 57.

**Results:**

Twenty-two patients received vandetanib (*n *= 10, 100 mg; *n *= 12, 300 mg). Baseline measurements of iAUC_60 _and K^trans ^were reproducible, with low intrapatient coefficients of variation (11% and 24%, respectively). Estimates of mean % changes from baseline were -3.4% (100 mg) and -4.6% (300 mg) for iAUC_60_, and -4.6% (100 mg) and -2.7% (300 mg) for K^trans^; these changes were not significantly different between doses. The exploratory T2* measurement showed a significant increase at 300 mg versus 100 mg (*P *= 0.006). Both doses of vandetanib were generally well tolerated; common toxicities were fatigue, rash and diarrhea (majority CTC grade 1 or 2). The pharmacokinetic profile of vandetanib was similar to that observed previously. There were no RECIST-defined objective responses; five patients experienced stable disease ≥8 weeks.

**Conclusion:**

In this study in patients with advanced colorectal cancer, vandetanib did not modulate gadolinium uptake in tumor vasculature and tissue measured by the DCE-MRI parameters iAUC_60 _and K^trans^.

**Trial registration:**

NCT00496509 (ClinicalTrials.gov); D4200C00050 (AstraZeneca)

## Background

Vascular endothelial growth factor (VEGF) has a pivotal role in tumor angiogenesis, which is required for the growth of most solid tumors and the formation of metastases. The VEGF signaling pathway is a validated therapeutic target in several solid tumors, including advanced colorectal cancer [1], non-small-cell lung cancer [2] and renal cell carcinoma [3,4].

Dynamic contrast-enhanced magnetic resonance imaging (DCE-MRI) is a non-invasive functional imaging technique that permits indirect measurement of tumor hemodynamics. It may therefore be suitable for monitoring the effects of VEGF signaling inhibitors on tumor vasculature. DCE-MRI utilizes a low molecular weight paramagnetic contrast agent such as gadolinium-DTPA, which readily diffuses from the blood to the extravascular extracellular space. By acquiring a set of rapid MR images, the time course of the change in T1 relaxation time induced by the contrast agent may be followed. Contrast agent concentration can be calculated from T1 relaxation times using the known linear relationship [5]. The time course obtained can be characterized by the initial area under the contrast agent concentration-time curve (iAUC) or a pharmacokinetic model may be applied. With the latter, the data are fitted to estimate the transfer of contrast agent between the plasma and the extracellular, extravascular space (the transfer constant K^trans^). Although iAUC and K^trans ^are incompletely validated endpoints that are sensitive to changes in a number of hemodynamic parameters, including blood flow, blood volume, vessel permeability and vessel surface area [6], emerging data from several early-phase clinical trials of VEGF signaling inhibitors have shown changes in K^trans ^and/or iAUC that are consistent with reductions in VEGF-dependent tumor perfusion and vascular permeability [7-11].

Vandetanib (ZACTIMA™) is a once-daily oral anticancer drug that selectively targets VEGFR-dependent tumor angiogenesis and REarranged during Transfection (RET)- and epidermal growth factor receptor (EGFR)-dependent tumor cell proliferation and survival. Preclinical DCE-MRI studies of vandetanib have demonstrated acute effects on hemodynamic variables in human prostate and colon xenograft models consistent with inhibition of VEGF signaling [12,13]. Vandetanib is currently in phase III development in advanced non-small-cell lung cancer (NSCLC) and medullary thyroid cancer. Two doses of vandetanib were selected for investigation in the present study (100 mg and 300 mg). Previous phase I studies of vandetanib have shown these doses to be well tolerated and to achieve steady-state plasma levels that are likely to be biologically active [14-16]. In addition, both doses were clinically active as monotherapy in phase II studies in NSCLC [17] and medullary thyroid cancer [18,19].

The primary objective of this open-label, randomized phase I study (study code D4200C00050) was to assess by DCE-MRI the effect of once-daily vandetanib on K^trans ^and iAUC_60 _(iAUC of the first 60 s after contrast agent arrival) in patients with advanced colorectal cancer and liver metastases. An exploratory objective was to investigate the effects of vandetanib on the tumor by intrinsic susceptibility MRI, a technique that may have utility in measuring tumor hypoxia in response to vascular disruption [20].

## Methods

### Patients

Eligible patients were adults with histologically confirmed metastatic colorectal adenocarcinoma (stage IV) with at least one measurable hepatic lesion ≥20 mm, WHO performance status 0-2, life expectancy ≥12 weeks, and no significant cardiac, hematopoietic, hepatic and renal dysfunction. Patients with brain metastases were eligible if treated at least 4 weeks before the start of study treatment and if clinically stable without steroid treatment for ≥10 days. Key exclusion criteria were previous chemotherapy and/or radiotherapy (excluding palliative radiotherapy) less than 4 weeks before the start of study therapy, a QTc interval ≥480 ms during ECG screening, and poorly controlled hypertension. Patients for whom MRI scanning is contraindicated (e.g. pacemaker, heart valve replacement) were also excluded.

### Study design

In this open-label study, 24 patients were planned to be randomized 1:1 to receive once-daily oral doses of vandetanib 100 mg or 300 mg. There was no stratification and patients continued treatment until progressive disease, withdrawal due to toxicity, patient lost to follow up, severe non-compliance with the protocol or voluntary discontinuation by the patient. The primary objective of this study was to assess by DCE-MRI the effect of once-daily dosing with vandetanib on the tumor vasculature by determining iAUC_60 _and K^trans^. Secondary assessments included safety and tolerability, pharmacokinetics, and a preliminary evaluation of efficacy. Exploratory assessments included the effects of vandetanib on the tumor by intrinsic susceptibility MRI, measurement of the target tumor size by MRI, and the effect of vandetanib on soluble markers of angiogenesis.

The trial was approved by the Bundesinstitut für Arzneimittel und Medizinprodukte institutional review board/research ethics committee, and was conducted in accordance with the Declaration of Helsinki, Good Clinical Practice and the AstraZeneca policy on Bioethics. All patients provided written informed consent.

### Assessments

#### MRI

DCE-MRI and intrinsic susceptibility MRI (T2*) scans were performed during the same scan session. To obtain baseline and reproducibility measurements, two scans were performed within 14 days before the start of vandetanib treatment (day 1) and the minimum time between scans was 1 day. Subsequent scans were performed on days 2, 8, 29 and 57. All DCE-MRI data were acquired using a 1.5 T system (Magnetom Sonata, Siemens, Germany). For the dynamic scan, a time series of inversion recovery balanced SSFP (TrueFISP) images in one coronal slice (d = 10 mm) cutting the liver target lesion were acquired (α = 40°, TE = TR/2 = 1.24 ms). To obtain absolute T1-relaxation rates at each time point of the time series, images at seven inversion times after each inversion pulse were used [21,22]. A dose of 0.1 mmol/kg Gd-DTPA (Magnevist, Schering) was administered (3 mL/s) in a peripheral vein using a contrast agent power injector (Spectris, MEDRAD Inc.). To obtain a baseline measurement without contrast agent, the measurement started 36 s before contrast agent administration. Altogether the dynamic changes were determined for a period of 5 min 30 s with a temporal resolution of 3 s. The data obtained were used to compute the change in contrast agent concentration over time. The concentration curve was then fitted to obtain K^trans ^(the volume transfer constant between blood plasma and extravascular, extracellular space for contrast agent over the tumor region of interest [ROI]) [23]. The iAUC_60 _was calculated over the tumor ROI according to Evelhoch [24]. The ROIs were drawn and semiautomatically tracked to all images of the time series. The outline and tracking was checked by a second person. The mean signal over the ROI was used as input for the analysis. The longest diameter of the target lesion evaluated by LD_DCE-MRI _was measured using anatomical multi-slice transversal T1-w and T2-w MRI scans obtained as part of the MRI acquisition protocol. The area of the target lesion evaluated by DCE-MRI was also measured as part of the assessment. The reference lesions for the DCE-MRI analysis were chosen by a radiologist at the screening. The lesion had to be larger than 2 cm (longest diameter in plane), clearly definable and not necrotic. Intrinsic susceptibility MRI consisted of a multi-gradient echo sequence (TR = 65 ms; TE = 5.1-58.9 ms; 12 echoes) acquired before contrast agent administration and was used to determine T2* (effective transverse relaxation time).

#### Efficacy

A preliminary assessment of efficacy was measured by objective response rate and progression-free survival (PFS) based on Response Evaluation Criteria in Solid Tumors (RECIST). RECIST assessments were performed by contrast-enhanced computed tomography (CT) at baseline, day 57 and every 8 weeks thereafter. Subjects who had not progressed or died at the time of analysis were censored at the time of their latest assessment.

#### Safety and tolerability

Adverse events were reviewed at each scheduled visit and graded according to the National Cancer Institute Common Terminology Criteria for Adverse Events (CTCAE) version 3. The possible relationship of an adverse event to study treatment was assessed by the investigator. Twelve-lead ECGs were performed during screening, pretreatment (day 1), days 8, 15, 29, 57 and every 3 months thereafter. Criteria for prolongation of the QTc interval were clearly defined in the protocol. Patients who continued to receive vandetanib beyond day 57 were anticipated to attend follow-up visits every 4-6 weeks.

#### Blood sampling

To evaluate the pharmacokinetics in this patient population, blood samples collected pre-dose on day 1, pre-dose and 4-8 h post-dose on days 8, 15 and 29, and pre-dose and at 4, 6, 8 and 24 hours post-dose on days 2 and 57 were used to determine the plasma concentrations of vandetanib. The binding of vandetanib to plasma proteins was also determined. Plasma concentrations of vandetanib and the concentrations in plasma ultra-filtrate were determined using reverse-phase liquid chromatography and detection by tandem mass spectrometry. Blood samples collected during screening and pre-dose on days 1, 2, 8, 15, 29 and 57, and at withdrawal were used to determine levels of VEGF, EGFR, sVEGFR-2, tunica interna endothelial cell kinase (Tie2), basic fibroblast growth factor (bFGF), Angiopoietin-1 (Ang1) and Ang2. VEGF and bFGF were measured in EDTA-plasma samples and the remaining markers measured in serum as described previously [7].

### Statistical analyses

The effect of vandetanib on MRI parameters was assessed using repeated measures analysis of variance (ANOVA) model fitted to log_e _transformed variables, with baseline as a covariate, dose and visit as fixed effects, and subjects as a random effect. Comparisons were performed to provide the least squares estimates and corresponding 95% CIs at each visit. Results are reported as the mean percentage change and associated 95% CI from baseline by dose. The proportion of patients with a >40% reduction post-baseline for K^trans ^and iAUC_60 _has been summarized for each dose level; the >40% threshold was predefined and has been used previously for detection of anti-vascular activity by DCE-MRI [7]. One-sided *P *values were calculated for dose comparison of percentage decreases from baseline in K^trans^, iAUC_60 _and LC_DCE-MRI_. The effect of vasoactive agents on T2*, and whether this produces an increase or a decrease of T2*, depends on the balance between any change of blood volume and blood flow coupled with any change in oxygen utilization [25]. Since this effect could not be predicted in the present study, a two-sided *P *value was calculated for T2*. Population pharmacokinetic and pharmacokinetic-pharmacodynamic modeling was conducted using NONMEM software [26,27].

## Results

### Patients

From 15 August 2006, 22 patients were enrolled in two centers in Germany and received study treatment; 10 patients were randomized to the vandetanib 100 mg group and 12 patients to the vandetanib 300 mg group (Table 1). The analysis population consisted of all subjects who had received at least one dose of vandetanib (intent-to-treat [ITT]). Eighteen patients continued study treatment until progression, three patients discontinued (two due to an adverse event, one due to violation of exclusion criteria) and one patient was ongoing on vandetanib 300 mg at data cut-off (22 June 2007). Median exposure to vandetanib was 34 days (range 28-58) in the 100 mg group and 60 days (range 9-202) in the 300 mg group. The demographic characteristics and previous anticancer treatments were generally well balanced between the two cohorts, although there were more female patients in the vandetanib 300 mg group than in the 100 mg group.

**Table 1:** Patient characteristics (ITT population)

Baseline characteristics	Vandetanib 100 mg (*n *= 10)	Vandetanib 300 mg (*n *= 12)
Median age, years (range)	62.5 (38--77)	61 (41--73)
Male (%)	6 (60)	5 (42)
Female (%)	4 (40)	7 (58)
Race		
Caucasian (%)	10 (100)	12 (100)
WHO performance status (%)		
0 1	6 (60) 4 (40)	8 (67) 4 (33)
Previous chemotherapy regimens (%)		
Any	10 (100)	12 (100)
1	1 (10)	4 (33)
2	3 (30)	2 (17)
3 or more	6 (60)	6 (50)
Prior cetuximab therapy (%)	7 (70)	7 (58)
Prior bevacizumab therapy (%)	5 (50)	4 (33)

### MRI results

#### Primary variables

**Figure 1:**
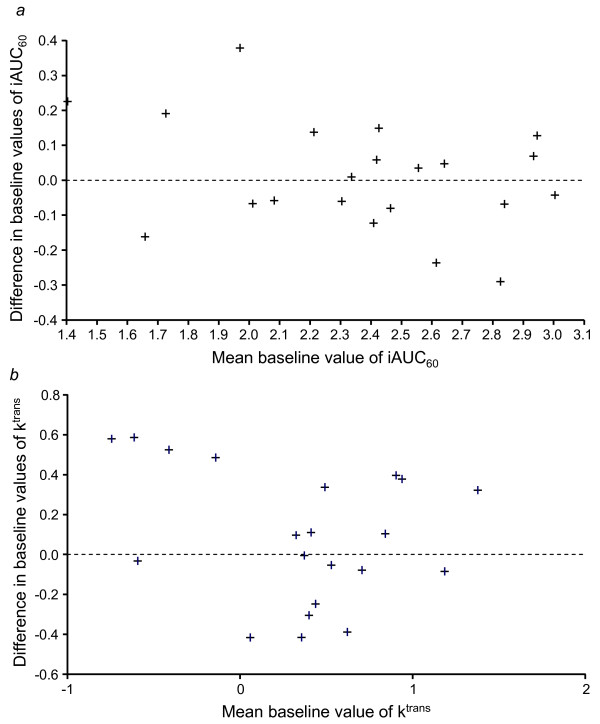
**Bland-Altman plot comparing initial and second baseline values for (a) iAUC_60 _and (b) K^trans^**. The difference between baseline values and mean baseline values is based on logarithmically transformed data.

**Figure 2:**
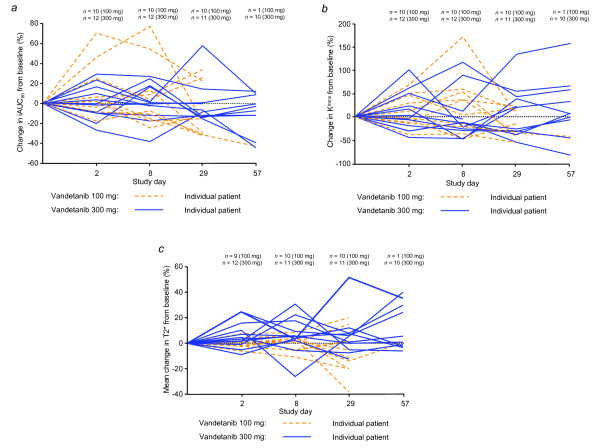
Estimated percentage change from baseline in (a) iAUC_60_, (b) K^trans^, and (c) T2* mean.

**Figure 3:**
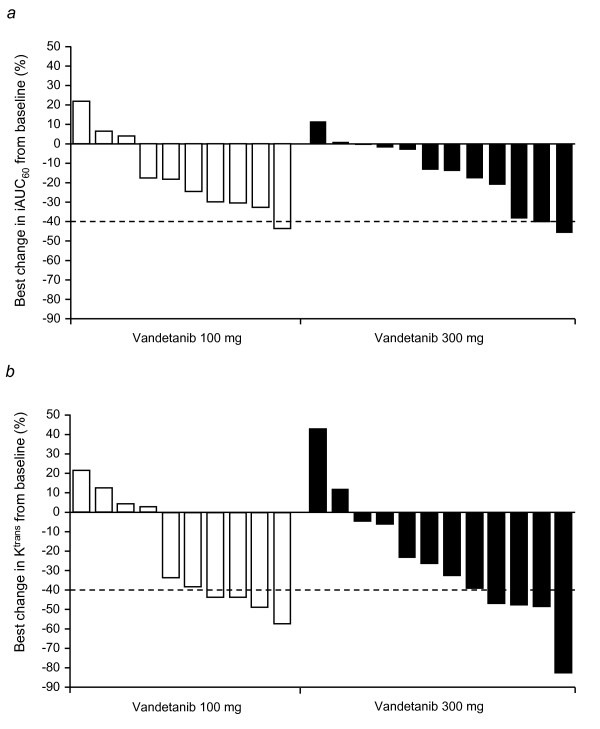
**Best percentage change from baseline in (a) iAUC_60 _and (b) K^trans^**. The best percentage change is defined as the biggest decrease, or smallest increase if no decrease. The threshold of activity was considered to be 40% (dashed line).

**Figure 4:**
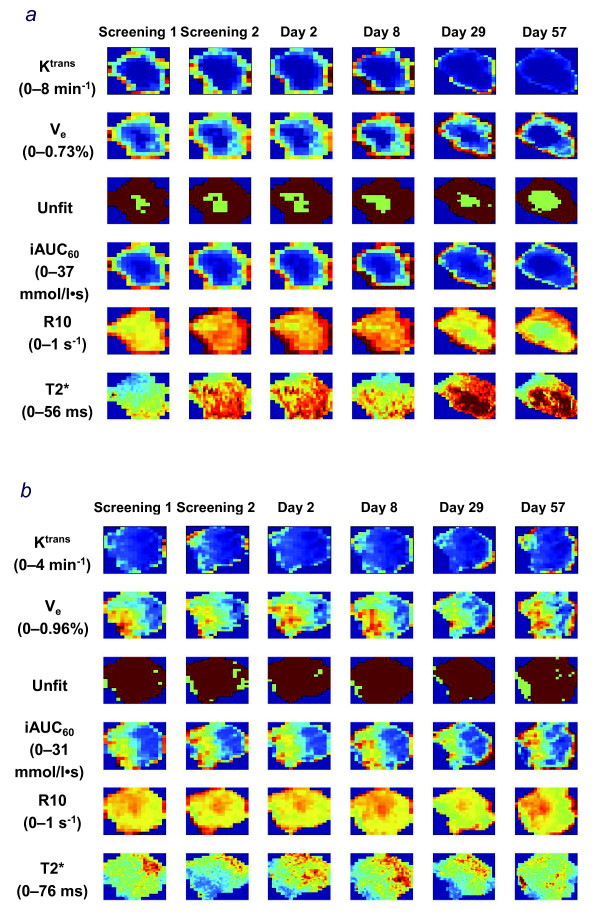
**Example parameter maps calculated from MRI data**. Both patients received vandetanib 300 mg and had RECIST-defined progressive disease on day 57 (patient 1983, Panel a) and day 62 (patient 2999, Panel b). Only the cutout of the lesion is shown. Definitions: K^trans ^volume transfer constant between blood plasma and extravascular extracellular space V_e_, extravascular extracellular volume fraction; Unfit image pixels, which could not be fitted by DCE-MRI models (low uptake of contrast agent); iAUC_60 _initial area under the DCE-MRI contrast agent concentration--time curve after 60 s; R10 native longitudinal relaxation rate constant before contrast agent administration;T2* effective transverse relaxation time.

An assessment of the within-patient variability in iAUC_60 _and K^trans ^revealed that these baseline DCE-MRI measurements were reproducible, with low estimated intrapatient coefficients of variation (11.3%, iAUC_60_; 24%, K^trans^). While differences were identified between the two baseline measurements for both parameters, Bland-Altman plot analyses supported the definition of baseline as the average of the two baseline measurements (Fig. 1a and 1b). The magnitude of change in either iAUC_60 _or K^trans ^was not significantly different between the vandetanib 100 mg and 300 mg cohorts (Table 2). The mean % changes from baseline in iAUC_60 _and K^trans ^showed small reductions in both treatment groups (Table 2; Fig. 2a and 2b). The best change from baseline in iAUC_60 _and K^trans ^for each patient is shown in Fig. 3a and 3b, respectively. One patient in each cohort showed at least once a >40% reduction from baseline in iAUC_60_. Four patients in each cohort showed at least once a comparable decrease of >40% in K^trans^. Consecutive decreases of >40% were not observed in any patients for iAUC_60 _and in only two patients for K^trans^. Fig. 4 illustrates composite MRI parametric images.

**Table 2:** Mean % change from baseline in MRI parameters (full analysis set)

	Mean % change from baseline (95% confidence interval)		*P *value* (300 mg vs. 100 mg)
		
	Vandetanib 100 mg	Vandetanib 300 mg	
Primary variables			
iAUC_60_	--3.4 (--13.6, 8.1)	--4.6 (--13.4, 5.0)	0.429 (one-sided)
K^trans^	--4.6 (--22.4, 17.4)	--2.7 (--18.4, 16.2)	0.558 (one-sided)

**Exploratory variables**			

T2*	--2.2 (--7.1, 2.9)	7.3 (3.1, 11.7)	0.006 (two-sided)
LD_DCE-MRI_	16.1 (9.7, 22.9)	8.0 (2.9, 13.4)	0.029 (one-sided)

iAUC_60 _initial area under the DCE-MRI contrast agent concentration--time curve after 60 s

K^trans ^volume transfer constant between blood plasma and extravascular extracellular space

T2* effective magnetic transverse relaxation time

LD_DCE-MRI _length of longest diameter of target lesion measured as part of the DCE-MRI acquisition protocol

*The effect of vandetanib on MRI parameters was assessed using repeated measures ANOVA, which was fitted to the log-transformed baseline as a covariate and dose as a factor. Point and interval estimates were exponentially back transformed to provide the estimates of the % differences. The preplanned statistical comparisons were between the dose levels and not within dose levels. Only the least squares mean (95% CI) changes are reported within dose. See 'Statistical analyses' section for full details

#### Exploratory variables

Mean T2* was measured as a function of tumor oxygenation using intrinsic susceptibility MRI. Deoxyhemoglobin creates a large magnetic disturbance next to blood vessels inducing signal loss on MR images which can be quantified by T2* shortening. Therefore T2* can be used to monitor changes in the concentration of deoxyhemoglobin, whether this is caused by fractional desaturation of oxygen from red blood cells or blood flow alterations. In the absence of any change of blood volume, agents that decrease blood flow and oxygenation may therefore decrease T2*. Baseline T2* measurements were reproducible, with a low intrapatient coefficient of variation (12.5%). Analysis of the mean change in T2* from baseline revealed a dose effect; the increase in T2* in the 300 mg cohort was significantly different from the small decrease observed in the 100 mg cohort (two-sided *P *= 0.006; Table 2, Fig. 2c). Similar results were obtained for median T2* (data not shown).

The length of the longest diameter of target lesion (LD_DCE-MRI_) was recorded in the pre-contrast DCE-MRI scan. Analysis of the LD_DCE-MRI _data from days 2, 8, 29 and 57 showed mean increases from baseline in both cohorts. These increases were less pronounced in the 300 mg cohort, with evidence of a significant dose effect (one-sided *P *= 0.029; Table 2). A similar trend was also observed for the lesion area, although with a larger intrapatient co-efficient of variation, which was expected due to repositioning of the imaging slice between scans (data not shown).

### Pharmacokinetics

**Figure 5:**
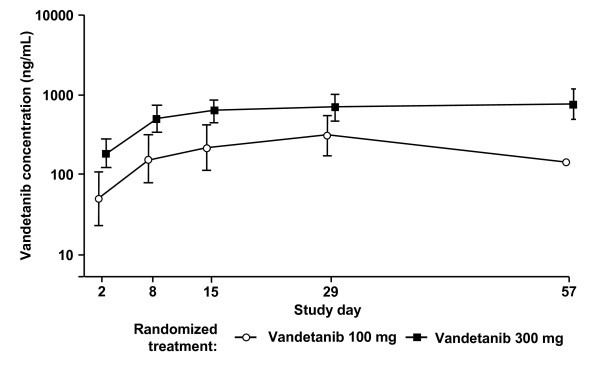
**C_min _plasma concentrations (ng/mL) for vandetanib 100 mg and 300 mg**. Data are shown as geometric mean (± SD).

After two doses of vandetanib, both the area under the curve to 24 h (AUC_0-24_) and the maximum concentration (C_max_) increased in a dose proportional manner, with gmean AUC_0-24 _of 1370 ng/mL·h (100 mg) and 4913 ng/mL·h (300 mg), and gmean C_max _of 72.7 ng/mL (100 mg) and 268.5 ng/mL (300 mg). The gmean accumulation at steady state was 4.3-fold in the 300 mg group and 6.12-fold for the one evaluable patient in the 100 mg dose group. Determination of C_min _throughout the study period showed that steady-state exposure was achieved from day 15 onwards (Fig. 5). The fraction of vandetanib unbound on day 2 was approximately 0.065 for both doses and, based on the 300 mg cohort, this was unaltered at the higher levels observed at steady state. A population pharmacokinetic-pharmacodynamic analysis showed little evidence of any correlation between the DCE-MRI variables and either the plasma concentration, daily exposure or total exposure to free or total vandetanib (data not shown).

### Soluble markers of angiogenesis/tumor activity

Higher plasma levels of VEGF were detected at both vandetanib doses following multiple dosing, although large variability was observed (data not shown). There was no suggestion of a dose effect. No consistent time- or dose-related changes from baseline were observed for the other markers evaluated (sVEGFR-2, bFGF, EGFR, Tie-2, Ang1 and Ang2; data not shown).

### Efficacy

**Figure 6:**
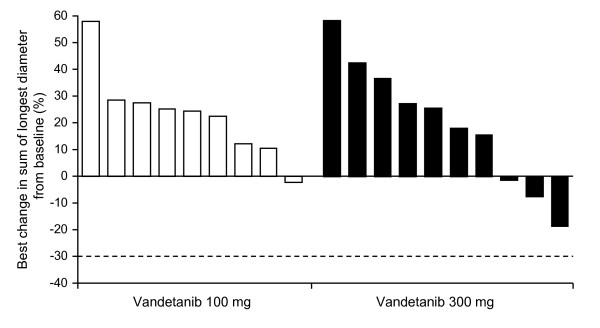
**Best percentage change from baseline in the size of target lesion (RECIST assessment performed by CT)**. The best percentage change is defined as the biggest decrease, or smallest increase if no decrease. The threshold for partial response is --30% (dashed line).

There were no RECIST-defined objective responses as assessed by contrast-enhanced CT. Among the 21 evaluable patients, five patients in the 300 mg group had a best response of stable disease ≥8 weeks and the remaining 16 patients experienced progressive disease. One patient in the 300 mg group had no post-baseline measurements and was therefore not evaluable. A waterfall plot of the best percentage change from baseline in the size of target lesions is presented in Fig. 6. Median PFS was 62 days (95% CI, 57 to 177) in the 300 mg group and 34 days (95% CI, 33 to 37) in the 100 mg group.

### Safety and tolerability

Both vandetanib doses were generally well tolerated. The most frequently reported adverse events, irrespective of causality, were fatigue, diarrhea, dry mouth and nausea (Table 3). More adverse events were reported in the 300 mg group compared with the 100 mg group, which is consistent with the greater number of days on treatment for the 300 mg group. The majority of adverse events were CTCAE grade 1 or 2, including all cases of diarrhea. The most common adverse events considered by the investigator to be related to vandetanib were dry mouth, dysphonia (both *n *= 5), diarrhea, fatigue, acne, dry skin (all *n *= 4) and hypertension (*n *= 3). Four of these adverse events were CTCAE grade 3 (all *n *= 1): allergic dermatitis, fatigue, photosensitivity reaction (all 300 mg) and hypertension (100 mg). No grade 4 events were reported. Adverse events that were considered by the investigator to be related to study treatment were mostly manageable by dose reductions or interruptions. Two patients in the 300 mg group experienced adverse events that led to discontinuation of treatment: allergic dermatitis and photosensitivity reaction (both grade 3) in one patient and QTc prolongation (grade 2) in another. Nine deaths occurred during this study before data cut-off and all were as a result of disease progression. Clinical laboratory evaluations did not show any clinically relevant changes in any clinical chemistry, hematology and urinalysis parameter. There was also no consistent trend in mean blood pressure values, although increases in systolic and/or diastolic blood pressure were observed during treatment, particularly in patients with a history of hypertension or patients who were borderline hypertensive at study entry. These increases in blood pressure were considered by the investigator to be related to vandetanib.

**Table 3:** Adverse events reported in >3 patients overall

Adverse event*	Vandetanib 100 mg (*n *= 10) *n *(%)	Vandetanib 300 mg (*n *= 12) *n *(%)	Total (*n *= 22) *n *(%)
Fatigue	6 (60)	7 (58)	13 (59)
Rash^†^	2 (20)	10 (83)	11 (50)
Diarrhea	2 (20)	7 (58)	9 (41)
Dry mouth	2 (20)	4 (33)	6 (27)
Nausea	3 (30)	3 (25)	6 (27)
Anorexia	3 (30)	2 (17)	5 (23)
Dysphonia	1 (10)	4 (33)	5 (23)
Abdominal pain	1 (10)	3 (25)	4 (18)
Acne	0	4 (33)	4 (18)
Cough	1 (10)	3 (25)	4 (18)
Dizziness	3 (30)	1 (8)	4 (18)
Dry skin	1 (10)	3 (25)	4 (18)
Flatulence	1 (10)	3 (25)	4 (18)
Hypertension	1 (10)	3 (25)	4 (18)
Insomnia	3 (30)	1 (8)	4 (18)
Nasopharyngitis	2 (20)	2 (17)	4 (18)
Peripheral edema	4 (40)	0	4 (18)
Sinus tachycardia	1 (10)	3 (25)	4 (18)
Weight decreased	1 (10)	3 (25)	4 (18)

*MedDRA-preferred term except for rash

^†^MedDRA grouped term, which includes the following preferred terms: dry skin, erythema, photosensitivity reaction, rash papular, rash pustular, dermatitis allergic, exfoliative rash, and rash erythematous

## Discussion

This randomized, open-label study used DCE-MRI to investigate the effect of once-daily oral dosing with vandetanib (100 mg or 300 mg) on tumor perfusion and vascular permeability in 22 patients with advanced colorectal cancer and liver metastases. The primary DCE-MRI variables of iAUC_60 _and K^trans ^did not show any statistically significant changes from baseline for either treatment group. Therefore, the study did not support the hypothesis that vandetanib has effects on tumor vasculature, as defined by changes in gadolinium uptake measured by iAUC_60 _and K^trans^. The safety and pharmacokinetic profiles of vandetanib were similar to those observed in previous phase I studies [15,16]. Both vandetanib doses were generally well tolerated with no new toxicities reported. A preliminary assessment of efficacy showed no RECIST objective responses in either treatment group, with five patients in the 300 mg group experiencing a best response of stable disease.

There are several possible explanations for the absence of detectable changes in gadolinium uptake and tumor shrinkage with vandetanib in this setting. Although variations in institutional DCE-MRI protocols and different patient populations do not permit direct comparison, studies of other VEGFR-2 tyrosine kinase inhibitors have demonstrated reductions in iAUC/K^trans ^in patients with advanced cancer [7-10]. Therefore, one explanation could be that vandetanib is not sufficiently active versus VEGFR-2 at the two doses investigated. However, this seems unlikely given that vandetanib has previously demonstrated single-agent antitumor activity at 100 mg and 300 mg in NSCLC [17] and in medullary thyroid cancer [18,19]; the present study also showed some evidence of antitumor effects (though not tumor shrinkage), with five patients in the 300 mg cohort experiencing stable disease. Inhibition of EGFR (NSCLC) and RET (medullary thyroid cancer) tyrosine kinases is also likely to be contributing to the activity of vandetanib in these tumor types; nevertheless, its relatively greater potency versus VEGFR-2 *in vitro *[14] suggests that vandetanib should achieve at least comparable inhibition of VEGFR-2 versus EGFR/RET *in vivo*. Moreover, in the present study, both vandetanib doses achieved steady-state plasma drug levels that were several-fold greater than the IC_50 _for inhibition of VEGF-dependent proliferation of human umbilical vein endothelial cells (29 ng/mL) [28]. An anti-VEGFR-2 effect of vandetanib at 100 mg and 300 mg is also supported by an exploratory pharmacodynamic study in patients with breast cancer, which showed inhibition of VEGFR-2 phosphorylation in skin biopsy tissue after 28 days of vandetanib treatment [29].

A second explanation may be that vandetanib is not active against the tumor vasculature in this particular disease setting. Indeed, the antitumor effects of vandetanib in this group of patients with colorectal cancer were modest compared with its single-agent activity in NSCLC [17] or medullary thyroid cancer [18,19]. Furthermore, the canonical changes in plasma VEGF and VEGFR-2 that have been observed with vandetanib in NSCLC [17] and with other VEGFR tyrosine kinase inhibitors across different tumor types [7,30] were not seen in the present study. In patients with colorectal cancer, objective tumor responses and effects on gadolinium uptake in tumor vasculature have been observed in single-agent studies of cediranib [7] and vatalanib [31]. Both of these VEGFR tyrosine kinase inhibitors, as well as bevacizumab, have activity versus VEGFR-1 and VEGFR-2 signaling [32,33]. In contrast, vandetanib is selective for VEGFR-2 versus VEGFR-1 [28]. It is known that colorectal tumor cells express VEGFR-1 and that autocrine signaling may play a role in tumor cell survival/migration [34]. Activity versus VEGFR-1 may therefore be an important contribution to any effects of antiangiogenic agents on both RECIST assessments and gadolinium uptake in colorectal cancer. In this respect, it is interesting that a recent pan-tumor study with CDP791, a high affinity PEGylated di-Fab conjugate that specifically binds VEGFR-2, showed limited efficacy and no effect on K^trans ^[35].

As discussed above, vandetanib has additional activity versus EGFR and the adverse event profile of vandetanib in this and previous studies [17,36,37] is consistent with pharmacodynamic inhibition of both VEGFR (hypertension) and EGFR signaling (rash, diarrhea). Combining inhibition of VEGF (bevacizumab) and EGFR (cetuximab) signaling on a background of chemotherapy has been investigated in two recent colorectal cancer studies, which produced different outcomes. The exploratory efficacy results from the BOND-2 study in irinotecan-refractory, bevacizumab- and cetuximab-naïve patients suggested that adding bevacizumab to cetuximab ± irinotecan may be more effective compared with historical controls [38]. However, the first-line CAIRO-2 study found that adding cetuximab to bevacizumab, capecitabine and oxaliplatin resulted in a significantly shorter PFS [39]. The CAIRO-2 authors speculated that these results may be due to a negative interaction between cetuximab and bevacizumab, and noted that the incidence of hypertension, a relatively common side effect of treatment with bevacizumab and other VEGF signaling inhibitors, was significantly reduced in patients receiving cetuximab. These data suggest, at least in some settings, that the vascular effects associated with VEGF inhibition may be diminished with concomitant EGFR inhibition. Other than vandetanib, AEE788 is the only dual VEGFR and EGFR tyrosine kinase inhibitor in clinical development and it is worth noting that AEE788 also showed no effect on gadolinium uptake in patients with advanced colorectal cancer and liver metastases [40]. An additional factor in the present study is that most patients had received previous treatment with bevacizumab and/or cetuximab, which may have affected responsiveness to subsequent VEGFR-2/EGFR tyrosine kinase inhibition. The mechanism of tumor resistance to the monoclonal antibodies bevacizumab and cetuximab is not well understood and warrants further investigation.

It is also possible that vandetanib treatment may induce hemodynamic changes, such as normalization/remodeling of the tumor vasculature as hypothesized by Jain [41], that would not necessarily be detected by estimating changes in K^trans ^and iAUC_60_. More complex DCE-MRI approaches such as the St Lawrence and Lee model [42], which is able to derive independent measurement of blood flow, blood volume and permeability surface area, may be more appropriate for detecting complex changes in tumor vascularity and hemodynamics. Normalization of the tumor vasculature might also be expected to improve tumor oxygenation and blood flow. In this regard, the results from the exploratory assessment of T2* using intrinsic susceptibility MRI merit discussion. Changes in T2* can be used to monitor changes in deoxyhemoglobin and an increase in T2* could result from improved tumor oxygenation and blood flow (i.e., normalization) [25]. However, T2* is influenced by other factors and is therefore a difficult parameter to interpret on its own [25,43]. In the absence of detectable effects on tumor hemodynamics as measured by DCE-MRI, an increase in T2* could be attributed to an increase in tumor cell death [43,44]. As such, the significant increase in T2* at vandetanib 300 mg compared with 100 mg in the present study may reflect increased tumor necrosis at the higher dose. Further correlative work is needed to understand the biological basis of changes in T2* in the clinical setting.

Population pharmacokinetic-pharmacodynamic analyses showed no correlation between vandetanib exposure and any of the pharmacodynamic parameters analyzed. Given the long half-life of vandetanib, it may take up to 4 weeks for vandetanib to reach steady state [14]; in the present study, steady state was attained from day 15 at the earliest, but was mostly from day 22 onwards. It is not fully understood how tumor growth/adaptation during this prolonged period of drug accumulation may affect pharmacodynamic variables.

## Conclusion

In the present study, DCE-MRI assessments of iAUC_60 _and K^trans ^provided no evidence that vandetanib modulated gadolinium uptake within the tumor vasculature of patients with advanced colorectal cancer and liver metastases. As discussed, these findings from a small open-label study of only 24 patients should be interpreted with caution, particularly since vandetanib has previously demonstrated evidence of antitumor activity in phase II studies in advanced NSCLC and medullary thyroid cancer that is consistent with inhibition of VEGFR activity. Vandetanib is one of a number of VEGF signaling inhibitors in clinical development and each has a different pharmacological profile [45]. We raise the possibility that the different selectivity profiles could result in agent-specific pharmacodynamic effects on tumor vasculature that may not be completely accounted for by changes in DCE-MRI variables such as iAUC_60 _and K^trans^. Vandetanib continues to be investigated in a range of other tumor types, including colorectal cancer and phase III programs in advanced NSCLC and medullary thyroid cancer.

## Competing interests

TPM, JT, ADK and AJR are all full-time employees of AstraZeneca. The authors declare no other competing interests.

## Authors' contributions

All authors contributed to the design of the study or manuscript writing, and have read and approved the final manuscript.
